# Bulletin of the Buffalo General Hospital

**Published:** 1911-06

**Authors:** 


### Social Service Department

Miss Lucy W. Stockton in charge of the social service department of the hospital has made the following report:

During the months of March and April fifty-four new cases were reported to the department. In addition forty-five old cases have been active during the two months, making a total of ninety-six cases. The new cases have been referred from the following sources:

Maternity ward, 5; Children's ward, 4; Female medical, 5; Female surgical, 6; Male, medical, 3; Male surgical, 3; House (B.G.H.), 2; Outside, 3; Outside for visiting and reports only, 23; Total, 54.

These cases have been referred for the following reasons:

I. Care after discharge: Investigation of home conditions, 9; Transfer to other institutions, 4; Change of occupation, 2; Convalescent care, 2.

II. Temporary home and work, 3; (a) Temporary home, 1; (b) Work, 2.

III. Unmarried mothers, 3.

IV. (a) In wards only, 2; (b) In homes only, 1; (c) Visiting in wards and report, 23.

V. For loans, 2. Total, 55.

In caring for the patients in March and April we have worked with the following agencies and institutions:

Charity Organization Society, 1; Superintendent of Poor's Office, Children's Department, 2; Erie County Home, 1; Erie County Hospital, 1; Erie County Lodging House, 1; Dispensary, Michigan and Broadway, 1; High Street Dispensary, 3; Overseer of the Poor, 2; Women's Educational and Industrial Union, 1; Rector of St. James Episcopal Church, 1; Visiting Catholic Priest, 1; Dr. Arthur, Superintendent Gowanda State Hospital, 1; Polish Charity Circle, 3; Ingleside Home, 1; Rescue Mission, 1; Salvation Army Home, 1; Dodge Street Catholic Orphan Asyslum, 1.

Visits made in wards, 808; Visits to homes, 104; Other visits made, 175; Visits of patient to office, 32; Visits about patients to office, 58; Visits of others to office, 29.

---

### Training School Graduation

The training school graduating exercises will be held on June 12 at 8 o'clock in the nurses' gymnasium and the following will receive diplomas:

Harriet A. Manley, Sarah H. Pettit, Agnes A. Johnston, Julia A. Smith, Blanche H. Schaffer, Berna Magee, Minnie Twamley, Mary E. Young, Mina Ryckman, Louise Kenyon, Grace E. Edwards, Elizabeth Gallup, Minnie Wilkinson, Inga C.

Glerum, Eva Fenwick, Alice E. Buse, Margaret B. Miller, Mabel B. Borden, Floretta M. Williamson, Maude Caplan.

Diplomas will be presented by Mr. Charles W. Pardee, president of the hospital; Dr. J. W. Putnam will present the badges. The address of the evening will be delivered by Rev. William H. Boocock, instructor in religious education at the First Church. Music will be furnished by Kuhn's orchestra and a double male quartette. There will be dancing.

---

### Practical Demonstration of Nursing

On the afternoon of May 12, the graduating class of the training school gave a demonstration of nursing in the clinic at the hospital before an audience which filled the amphitheater to its capacity. Dr. Roswell Park presided and prefaced the work of the nurses by an address in which he outlined the history of hospital work and showed how greatly present-day methods were responsible for the work of such men as Fliedner and such nurses as Florence Nightingale and Clara Barton. He traced the evolution of nursing from the crudities of its early days to the marvelous perfection of today. Referring to the Buffalo General Hospital of the present, Dr. Park showed how it had grown from a capacity of less than a dozen beds to three hundred and seventy-five and that its perfection of management had made it possible for the institution to care for three hundred and seventy-five patients at one time. He emphasised the wide range of accommodation from the free beds in the wards to the private rooms with every personal convenience; he referred to the wonderful improvement in hospital methods such, for instance, as the establishment of the Pardee laboratory, which gives the surgeon and physician accurate pathologic reports of their cases within a very short time after admission. Dr. Park then referred to the generosity of the Gates family upon which is builded the bulk of the improvements in the hospital—and even the building itself. The clinic and its fittings was the gift of this generous family and an idea of the great benefit humanity had received as a result was given when he stated that almost 15,000 operations had been performed under the best possible conditions making for the patients' safety and comfort.

The program for the demonstration was as follows:

1. (a) Two ways of changing the under-sheet of a patient who cannot be turned; left thigh broken; (b) First aid methods of transportation.

2. (a) Lifting a patient from the floor to the bed on an improvised stretcher; carrying the patient from one room to another. (b) Bandaging for dislocated shoulder.

3. (a) Turning a mattress with the patient in bed. (b) Bandaging for injury to head.

4. Bathing a baby.

For the first demonstration a ward bed was wheeled into the clinic bearing a patient with a supposed fracture of the thigh. The limb was splinted and bandaged and without moving the patient—to whom the slightest change of position was supposed to be exquisitely painful—the nurses changed the under sheet first from side to side and then from top to bottom. In these operations the patient was not moved. As the work of demonstration went forward Dr. Park described what was being done and assured the audience that none of the patients was a real patient; that the injuries were merely make-believe, but that the same care and the same methods were used as would be necessary under actual conditions. First aid methods of transportation such as are in use in the army and navy were demonstrated and then a patient supposedly severely injured was picked up in a blanket stretcher and carried to a bed. One of the most picturesque exhibitions was bandaging for a dislocated shoulder and the putting on of a cap bandage for covering the entire head. Rather a startling bit of work was that where a patient with the supposedly broken thigh had the mattress taken from under him, turned and replaced and the patient not taken from the bed. Then came the only real patient of the day, the baby who was to act as chief figure in the demonstration of bathing. It was a tiny little mite of humanity and, under the deft and careful handling of the nurse, was undressed, given a full bath, powdered, patted and primped and dressed and tucked away in its crib after its name tag had been replaced with a tiny blue ribbon. The use of the name tags in the hospital is a part of the daily routine. The first nursing demonstration was given last year and will be a feature of the graduation work of the senior class in the future.

### Statistical Record

For the first half of the fifty-third year of the hospital the statistical record is as follows:

| | | |
|---|---|---|
| Number of patients remaining October 1, 1910 | | 188 |
| New patients admitted during the six months | | 1,844 |
| Total treated | | 2.032 |
| Discharged: | | |
| Recovered | 1,060 | |
| Improved | 423 | |
| Unimproved | 104 | |

| | | |
|---|---|---|
| Otherwise discharged | 54 | |
| To other institutions | 13 | |
| Died | 128 | |
| Total discharged | | 1,728 |
| Remaining in hospital March 30, 1911 | | 350 |
| Total treated since opening of hospital | 65,828 | |
| Total day's relief first half fifty-second year | 34,123 | |
| Toal day's relief first half fifty-third year | 41,319 | |
| Daily average of patients fifty-second year | 187 plus | |
| Daily average of patients fifty-third year | 227 plus | |
| Largest number of patients, March 20, 1911 | 274 | |
| Ambulance calls | | 681 |

Time consumed on calls, 423 hours and 50 minutes.
Average time consumed on each call, 37 minutes.
Pardee Laboratory, first half of fifty-third year:

| | |
|---|---|
| Twenty-four hour samples of urine | 2,333 |
| Single samples | 2,195 |
| Stools | 330 |
| Leukocyte counts | 579 |
| Red counts | 160 |
| Hemoglobin estimations | 364 |
| Differential counts | 145 |
| Smears | 136 |
| Transudates and exudates | 30 |
| Vaccines | 55 |
| Iodine reactions | 23 |
| Gastric analyses | 159 |
| Sputa examinations | 219 |
| Blood and other cultures | 90 |
| Urine cultures | 60 |
| Polariscopic examinations | 26 |
| Special | 262 |
| Wassermann reactions | 74 |
| Total examinations | 7,240 |

REACTIONS TO TUBERCULIN.—Louis C. Rouglin, Atlanta, Ga., states that the study of reactions to tuberculin treatment is of the utmost importance. The cause of reaction is not fully explained. It is perhaps due to the introduction into the system of a foreign toxin, not attuned to the condition of the organism, which excites an increased irritability and activity of the toxin already present. A reaction may be local with swelling, redness, and the like, or it may be general with fever, pain in the limbs, and other general phenomena. It will last but a few hours or days. In treatment with tuberculin reaction should be avoided and a dose given which is below that needed to produce reaction.—*Medical Record,* 22, 1911.

